# Reward Processing in Attention-Deficit/Hyperactivity Disorder: A Meta-Analysis of Functional Magnetic Resonance Imaging Activation Studies

**DOI:** 10.1192/j.eurpsy.2025.529

**Published:** 2025-08-26

**Authors:** G. Zamora, E. Johnson-Venegas, C. Baten, A. Klassen, J. H. Shepherd, A. Catchpole, E. Davis, I. Dillsaver, C. Hunt, P. Hamilton, M. Sacchet, E. Woo, J. Miller, D. Hedges, C. Miller

**Affiliations:** 1 Psychology, California State University, Fresno, Fresno; 2 Brigham Young University, Provo; 3 Brigham Young University; 4 California State University, Fresno, Fresno, United States; 5 Biological and Medical Psychology, University of Bergen, Bergen, Norway; 6 Psychiatry, Massachusetts General Hospital, Harvard Medical School, Boston; 7 Psychology, Palo Alto University, Palo Alto; 8 Psychology, Brigham Young University, Provo, United States

## Abstract

**Introduction:**

Attention-deficit/hyperactivity disorder (ADHD) is one of the most prevalent mental disorders diagnosed in children and is characterized by complex, interacting symptoms. Although executive functioning systems are most frequently examined in functional magnetic resonance imaging (fMRI) activation studies of ADHD, atypical reward processing may also play a central role in ADHD and influence other symptoms, such as hyperactivity and impulsivity. This meta-analysis aims to advance our understanding of the neural basis of reward processing in ADHD, as measured by fMRI activation studies.

**Objectives:**

The present study aims to advance our understanding of the neural basis in reward processing in participants with ADHD by identifying aberrant functional activation in various brain regions compared with healthy controls.

**Methods:**

We conducted a comprehensive literature search in PubMed for whole-brain, task-based fMRI activation studies comparing participants diagnosed with ADHD to healthy controls in accordance with PRISMA guidelines. We then used multilevel kernel density analysis (MKDA) with ensemble thresholding (α = .05–.0001; FWE-corrected) to explore neural activation patterns associated with ADHD across all tasks and during reward processing tasks.

**Results:**

We obtained 57 primary studies (*N* = 4,366) that met our inclusion criteria. We found that patients with ADHD (*n* = 1,591), relative to healthy controls (*n* = 2,775), exhibited statistically significant (*p* < .005; FWE-corrected) differential activation in multiple brain regions of the cerebral cortex and basal ganglia, including robust effects across various tasks and task-specific effects observed during reward processing.

**Conclusions:**

These findings strengthen our understanding of the neural basis of reward processing in ADHD, which may inform new neurocognitive models of this heterogeneous disorder. Future studies should investigate disorder-specific and transdiagnostic neural features of ADHD and reward processing and explore clinical applications such as non-invasive brain stimulation and neurofeedback training.

**Disclosure of Interest:**

None Declared

